# Ultrahypofractionated Radiotherapy versus Conventional to Moderate Hypofractionated Radiotherapy for Clinically Localized Prostate Cancer

**DOI:** 10.3390/cancers14010195

**Published:** 2021-12-31

**Authors:** Hideya Yamazaki, Gen Suzuki, Norihiro Aibe, Daisuke Shimizu, Takuya Kimoto, Koji Masui, Ken Yoshida, Satoaki Nakamura, Yasutoshi Hashimoto, Haruumi Okabe

**Affiliations:** 1 Department of Radiology, Graduate School of Medical Science, Kyoto Prefectural University of Medicine, 465 Kajiicho Kawaramachi Hirokoji, Kamigyo-Ku, Kyoto 602-8566, Japan; gensuzu@koto.kpu-m.ac.jp (G.S.); a-ib-n24@koto.kpu-m.ac.jp (N.A.); dshimizu@koto.kpu-m.ac.jp (D.S.); t-kimoto@koto.kpu-m.ac.jp (T.K.); mc0515kj@koto.kpu-m.ac.jp (K.M.); 2 Department of Radiology, Kansai Medical University, Hirakata 573-1010, Japan; ken.yoshida@ompu.ac.jp (K.Y.); satoaki@nakamura.pro (S.N.); 3 Department of Radiology, Ujitakeda Hospital, Uji-City, Kyoto 611-0021, Japan; yasu06340829@yahoo.co.jp (Y.H.); h-okabe@takedahp.or.jp (H.O.)

**Keywords:** prostate cancer, ultrahypofractionation, late toxicity, radiotherapy

## Abstract

**Simple Summary:**

Recently, shortening treatment time is becoming more important. Ultrahypofractionated radiotherapy (UHF) for localized prostate cancer is a fascinating treatment strategy; however, the concept of a well-balanced, optimal dose during UHF radiotherapy remains a contentious strategy, with only a few studies on UHF already reported. We must wait for the results of randomized trials several years away. Therefore, we tried to reveal the acceptable schedule in comparison to conventional to moderate hypofractionated radiotherapy so far. We found that UHF using EQD2 ≤ 100 Gy_1.5_ is a feasible UHF schedule with a good balance between toxicity and efficacy.

**Abstract:**

The purpose of this study was to compare the toxicity (first endpoint) and efficacy (second endpoint) of ultrahypofractionated radiotherapy (UHF) and dose-escalated conventional to moderate hypofractionated radiotherapy (DeRT) for clinically localized prostate cancer. We compared 253 patients treated with UHF and 499 patients treated with DeRT using multi-institutional retrospective data. To analyze toxicity, we divided UHF into High-dose UHF (H-UHF; equivalent doses of 2 Gy per fraction: EQD2 > 100 Gy_1.5_) and low-dose UHF (L-UHF; EQD2 ≤ 100 Gy_1.5_). In toxicity, H-UHF elevated for 3 years accumulated late gastrointestinal and genitourinary toxicity grade ≥ 2 (11.1% and 9.3%) more than L-UHF (3% and 1.2%) and DeRT (3.1% and 4.8%, *p* = 0.00126 and *p* = 0.00549). With median follow-up periods of 32.0 and 61.7 months, the actuarial 3-year biochemical failure-free survival rates were 100% (100% and 100% in the L-UHF and H-UHF) and 96.3% in the low-risk group, 96.5% (97.1% and 95.6%) and 94.9% in the intermediate-risk group, and 93.7% (100% and 94.6%) and 91.7% in the high-risk group in the UHF and DeRT groups, respectively. UHF showed equivocal efficacy, although not conclusive but suggestive due to a short follow-up period of UHF. L-UHF using EQD2 ≤ 100 Gy_1.5_ is a feasible UHF schedule with a good balance between toxicity and efficacy.

## 1. Introduction

Prostate cancer became a major malignancy in developed countries [1]. As many curative treatment options exist, surgery, external beam radiotherapy, and brachytherapy, it is difficult to choose the best treatment option [2]. Recent advancements in radiotherapy for localized prostate cancer have enabled us to shorten the treatment period using hypofractionations and provide cost effectiveness and patient convenience. In the place of conventional 1.8–2-Gy fractionation, 2.3–3.4-Gy moderate hypofractionation has already become the standard of care [3]. Furthermore, ultrahypofractionation radiotherapy (UHF) of 5 Gy or more has emerged. The biological features of prostate cancer with a low α/β ratio also encouraged the adoption of these hypofractionations and UHF worldwide [2,3]. However, the concept of a well-balanced, optimal dose during UHF radiotherapy remains contentious, with only a few studies on the comparison of different UHF schedules already reported.

Ishiyama et al. reported an early experience of the safety and effectiveness of UHF in Japan using multi-institution data accumulation and created an open database for exploration [4,5,6]. We aimed to compare the outcomes of UHF with those of conventional to moderate hypofractionated radiotherapy. The superiority of high prescribed doses was confirmed with many randomized, controlled trials for localized prostate cancer [7] using biochemical failure-free survival (BFFS) rates. Then, the National Comprehensive Cancer Network (NCCN) Clinical Practice Guidelines in Oncology (2019) state that doses of 70 Gy or less in conventional fractions are not enough of a dose to treat intermediate- or high-risk prostate cancer [2]. Therefore, we set a control group that used dose-escalated radiotherapy (DeRT) with a prescribed dose of 72 Gy or more, which is equivalent to doses of 2 Gy per fraction (EQD2) (74 Gy for intermediate- to high-risk groups). To reduce bias, we used the propensity score analysis, including an inverse probability of treatment weighting (IPTW) method and propensity score-matched pair analysis. The aim of the present study was to compare the efficacy and especially toxicity of UHF and DeRT.

## 2. Materials and Methods

### 2.1. Patients

We retrospectively examined 253 patients treated with UHF (open data for public use) [6] and 499 patients treated with dose-escalated radiotherapy (DeRT) [270 Uji-Takeda Hospital (tomotherapy) and 229 open data]. The patient eligibility criteria were (1) patients treated with UHF or DeRT, (2) the clinical stage T1–T4 and N0M0 with histology-proven adenocarcinoma; the availability and accessibility of pretreatment data (initial prostate-specific antigen = iPSA) level, T classification, and the Gleason score sum (GS) to determine the stage according to the NCCN 2015 risk classification as follows: low (T1–T2a, GS 2–6, and iPSA < 10 ng/mL), intermediate (T2b–T2c, GS 7, or PSA 10–20 ng/mL), and high (T3–T4, GS 8–10, or PSA > 20 ng/mL) [1]. Exclusion criteria were (1) metastasis cases, (2) node-positive cases, and (3) EQD2Gy < 72 Gy (<74 Gy for intermediate- or high-risk categories) (Table 1). The purpose of this study was to compare the toxicity (first endpoint) and efficacy (second endpoint) of UHF and DeRT for clinically localized prostate cancer.

The definition of biochemical failure-free survival rate (BFFS) was the time from the initiation of radiotherapy to the date of biochemical failure and/or last follow-up, whichever came first, according to the Phoenix definition (nadir, +2 ng/mL) [2].

The toxicity analysis was performed with Common Terminology Criteria for Adverse Events version 4.0. The patients undergoing UHF (open data) and a part of those undergoing DeRT (open data) provided informed consent during the process of building public data, and all patients treated at Uji-Takeda hospital provided written, informed consent. We performed this study in accordance with the Declaration of Helsinki. We obtained the permission of the Institutional Review Board (Kyoto Prefectural University of Medicine: ERB-C-1403).

### 2.2. Treatment Planning

The detailed method of radiotherapy (UHF [4,5] or DeRT [6] in Uji-Takeda Hospital [8]) has been described elsewhere. All patients were treated with UHF or DeRT at various fractions (Table 2). According to previous studies, we divided the UHF group into two subgroups: H-UHF and L-UHF groups, using a cut-off value of EQD2 = 100 Gy_1.5_ (α/β = 1.5 Gy) [4,9].

In Uji-Takeda hospital, we used a 2.2 Gy/fraction schedule using D95 (95% of planning target volume (PTV) received at least the prescribed dose) of 74.8 Gy in 34 fractions (2.2 Gy/fraction) for intermediate- and high-risk patient, and 72.6 Gy in 33 fractions was used for low-risk cases, initially [8]. We changed the prescribed dose to 74 Gy (D95) in 37 fractions for the high- and intermediate-risk groups and 72 Gy in 36 fractions for the low-risk group (2 Gy/fraction). Between June 2007 and June 2009, we used a 2.2-Gy fraction schedule, which was changed to a modified 2-Gy fraction schedule from June 2009 to September 2013 [8].

### 2.3. Statistical Analysis

We used The R stat package [10] for the statistical analyses. Student *t*-tests were used for normally distributed data. Mann–Whitney U-tests for skewed data were used to compare means or medians, and percentages were analyzed using chi-square tests. The biochemical control rate, survival, and accumulated toxicity were analyzed using the Kaplan–Meier method, and comparisons were made using log-rank tests. We used Cox’s proportional hazards model for univariate and multivariate analyses of biochemical control rate. Statistical significance was set at *p* < 0.05. As we did not randomize the included patients, unbalanced baseline characteristics existed and could have led to a selection bias. Therefore, it influenced the decision to undergo UHF or DeRT. We used propensity score, which is defined here as the probability of being assigned to the DeRT or UHF group, given the patient characteristics. The logistic regression model was used in the calculation of the propensity scores, considering the baseline covariates shown in Table 2 (age, hormonal therapy history, T classification, GS, and pretreatment PSA). After the initial analysis of the whole cohort, we performed a propensity score-matched pair analysis to minimize the bias related to the choosing of and allocation to the DeRT or UHF groups. Five factors prescribed before were used to create a 1:1 matched cohort assigned to the UHF and DeRT groups.

## 3. Results

### 3.1. Patient and Tumor Characteristics

We examined 752 patients with stage T1–T4 N0M0 prostate cancer treated using UHF or DeRT. Table 1 presents the baseline patient characteristics of the UHF and DeRT groups. The median follow-up duration for the entire cohort was 47 months (range, 9–97 months) and the median patient age was 72 years (range, 51–86 years). The UHF group was used to treat patients with the earlier disease and less hormonal therapy history with shorter follow-up periods than those in the DeRT group. After matching, there were no significant differences for all variables used for matching (Supplemental Table S1).

### 3.2. Late Toxicity

The incidence of late gastrointestinal (GI) and genitourinary (GU) toxicities is shown in Table 3. The higher incidence of GU toxicities and equivalent incidence of GI toxicities occurred in the UHF group in comparison with the DeRT group. Among subgroups, the H-UHF group showed an elevated incidence of GI and GU toxicities compared to the DeRT and L-UHF groups.

**Figure 1:**
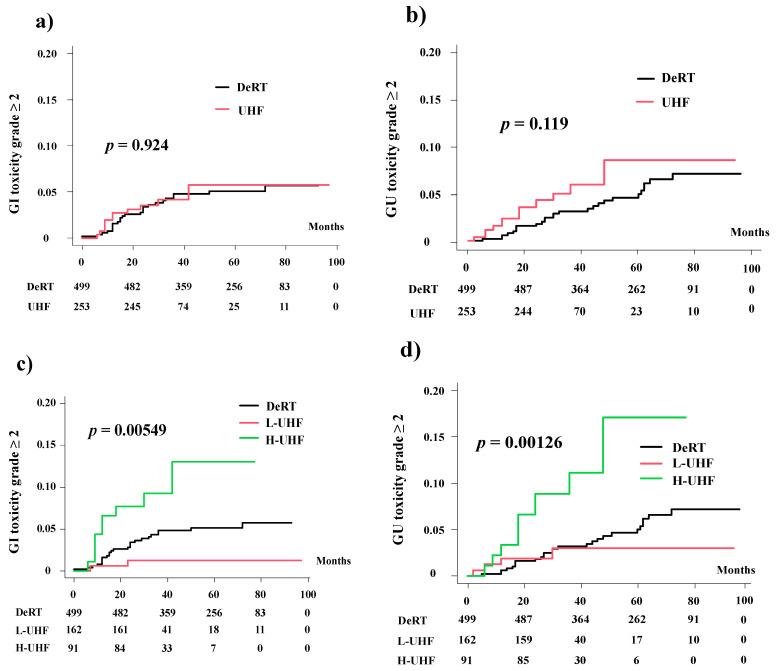
Comparison of accumulated incidence toxicity grade ≥ 2. (**a**) Accumulated incidence of grade ≥ 2 Gastrointestinal (GI) toxicity between DeRT and UHF. (**b**) Accumulated incidence of grade ≥ 2 Genitourinary (GU) toxicity between DeRT and UHF. (**c**) Accumulated incidence of grade ≥ 2 GI toxicity among three groups (DeRT vs. L-UHF vs. H-UHF). (**d**) Accumulated incidence of grade ≥ 2 GU toxicity among three groups (DeRT vs. L-UHF vs. H-UHF).

The 3-year cumulative incidence rate of grade ≥ 2 GI toxicities was 4.2% (5.8% at 5 years) in the UHF group and 4.8% (5.1%) in the DeRT group (*p* = 0.924; Figure 1). When the three groups were compared (UHF was divided into H-UHF and L-UHF), the 3-year and 5-year cumulative incidence rates of grade ≥ 2 late GI toxicities were 1.2% and 1.2% in the L-UHF group, respectively, 9.3% and 11.3% in the H-UHF group, respectively, and 4.8% and 5.1% in the DeRT group, respectively (*p* = 0.00549, Figure 1.The hazard ratios of H-UHF to DeRT and to L-UHF were 2.363 (95% CI: 1.094–5.1104, *p* = 0.0283) and 0.291 (95% CI: 0.069–1.236, *p* = 0.0944), respectively.

The 3-year cumulative incidence rate of grade ≥ 2 GU toxicities was 6% (8.6% at 5 years) in the UHF group and 3.1% (5% at 5 years) in the DeRT group (Figure 1, *p* = 0.119). In the three-group comparison, the 3-year (and 5-year) cumulative incidence rates of grade ≥ 2 late GU toxicities were 3% (3% at 5 years) in the L-UHF group, 11.1% (17% at 5 years) in the H-UHF groups, and 3.1% (5% at 5 years) in the DeRT group (*p* = 0.00126, Figure 1). The hazard ratios of H-UHF to DeRT and to L-UHF were 3.410 (95% CI: 1.596–7.283, *p* = 0.0015) and 0.757 (0.26–2.209, *p* = 0.6110), respectively.

### 3.3. Biochemical Control, Overall, and Prostate Cancer-Specific Survival

**Figure 2:**
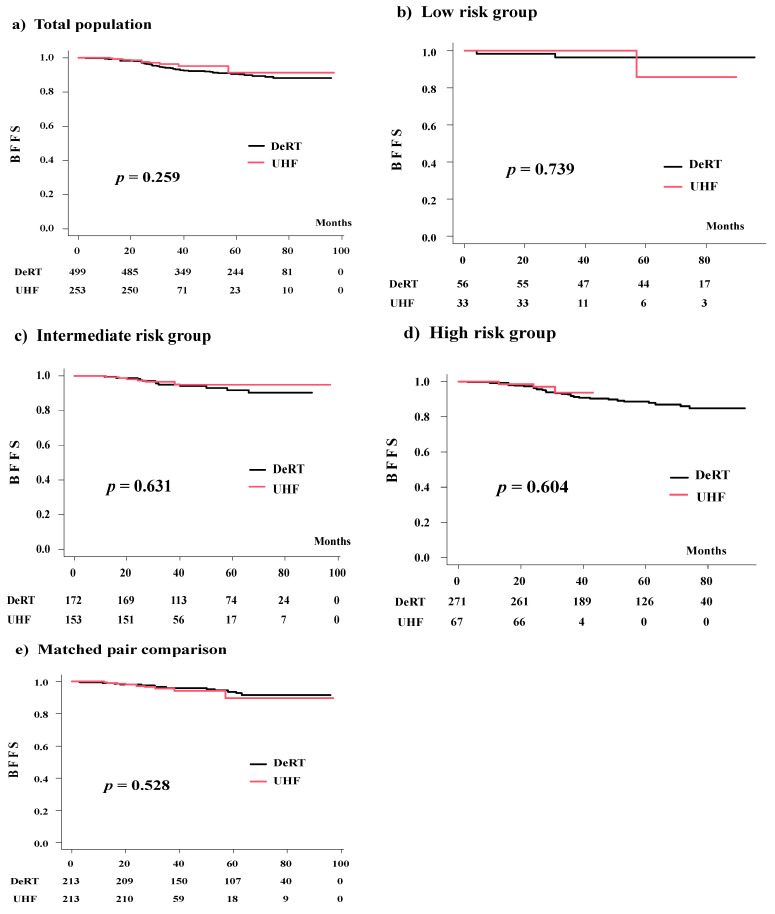
Biochemical control rates (BFFS) between UHF and DeRT.

The number of patients with biochemical failure was 45 in the DeRT group (9.01%) and 10 in the UHF group (3.95%). With median follow-up periods of 32.0 and 61.7 months, the actuarial 3-year BFFS rates were 96.3% (95% confidence interval [CI]: 92.7–98.2%) and 93.4% (95% CI: 90.7–95.1%) (*p* = 0.259) in the UHF and DeRT groups, respectively (Figure 1). The corresponding values were 100% and 96.3% (*p* = 0.739) in the low-risk group, 96.5% (95% CI: 91.7–98.5%) and 94.9% (95% CI: 90.1–97.4%) (*p* = 0.631) in the intermediate-risk group, and 93.7% (95% CI: 79.9–98.1%) and 91.7% (95% CI: 87.6–94.5%) (*p* = 0.604) in the high-risk group (Figure 2).

We generated a well-matched set of 213 patient pairs in each group. The actuarial 3-year BDFS rates were 95.6% (95% CI: 91.2–97.8%) and 95.9% (95% CI: 92–98%, *p* = 0.528, Figure 1d) in the UHF and DeRT groups, respectively. The hazard ratio was 1.321 (95% CI: 0.5547–3.147, *p* = 0.5293).

Table 4 shows the predictors of biochemical control on the univariate analysis including age (<75 vs. ≥75 years), T classification (≤T2 vs. ≥T3), the GS (≤7 vs. ≥8), and baseline PSA level (<10 vs. ≥10 ng/mL). On the multivariate analysis using Cox regression model, a high PSA level and hormonal therapy usage remained significant for improving biochemical control. The difference of treatment (DeRT or UHF) was not a significant factor for biochemical control.

**Figure 3:**
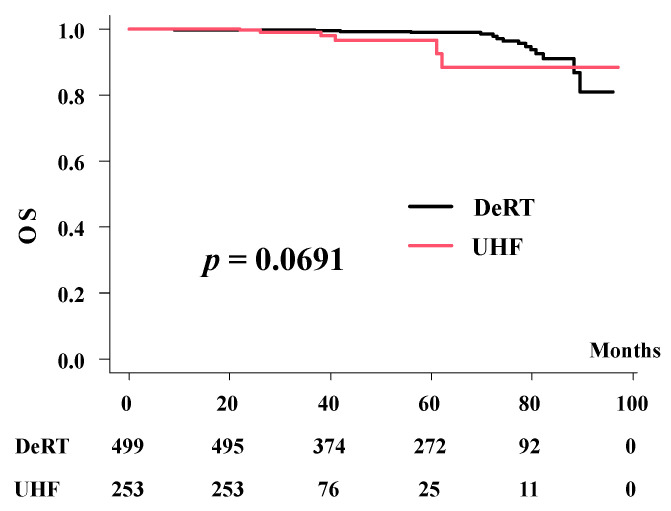
Overall survival rates between UHF and DeRT.

The 3-year and 5-year overall survival rates were 98% (95% CI: 93.1–99.4%) and 96.6% (95% CI: 90.1–98.8%) for UHF, and 99.8% (95% CI: 98.6–100%) and 98.9% (95% CI: 97.1–99.6%) for the DeRT groups (*p* = 0.0691)(Figure 3).

In the matched pair analysis, the 3-year and 5-year overall survival rates were 99.5% (95% CI: 96.7–99.9%) and 91.5% (95% CI: 72.5–97.6%) for UHF, and 100% and 99.4% (95% CI: 95.9–99.9%) for the DeRT groups (*p* = 0.285).

No prostate cancer-related deaths were observed in this cohort. The 5-year prostate cancer-specific survival rates were 100% in both the UHF and DeRT groups.

### 3.4. Comparison among the Three Groups (DeRT vs. L-UHF vs. H-UHF)

**Figure 4:**
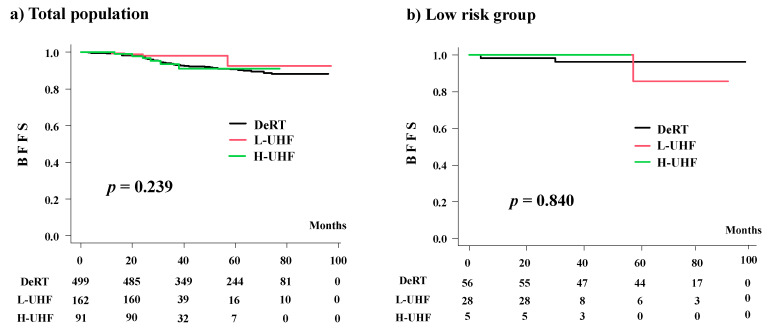

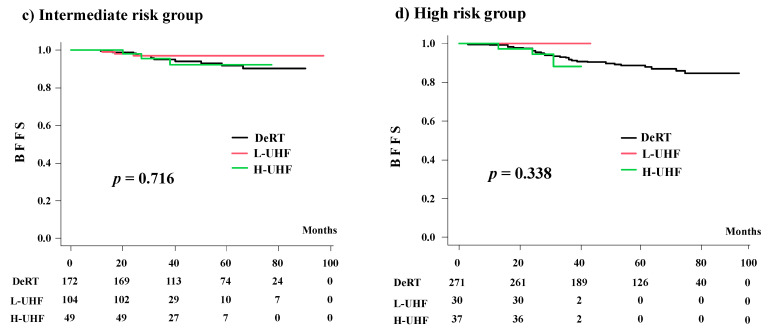
Biochemical control rates (BFFS) among three groups (L-UHF vs. H-UHF vs. DeRT).

A background comparison is shown in Supplemental Table S2. Advanced disease was treated in the H-UHF group compared to the L-UHF group. The number of patients who showed biochemical failure was four in the L-UHF group (2.46%) and six in the H-UHF group (6.593%) (*p* = 0.106). The actuarial 3-year BFFS rates were 98.1% (95% CI: 94.3–99.4%), 93.3% (95% CI: 85.5–97.0%), and 93.6% (95% CI: 85.1–97.3%, *p* = 0.239; Figure 2) in the L-UHF, H-UHF, and DeRT groups, respectively. The corresponding values were 100%, 100%, and 96.3% (95% CI: 85.9–99.1%) (*p* = 0.840) in the low-risk group; 97.1% (95% CI: 91.2–99.0%), 95.6% (95% CI: 83.3–98.9%), and 94.9% (95% CI: 90.1–97.4%) (*p* = 0.716) in the intermediate-risk group; and 100%, 94.6% (95% CI: 80.1–98.6%), and 91.7% (95% CI: 87.6–94.5%) in the high-risk group (*p* = 0.338) (Figure 3). Among all groups, the L-UHF showed equivalent outcomes compared to the H-UHF and DeRT groups (Figure 4).

## 4. Discussion

The recent HYPO-RT-PC randomized, control trial provided evidence that 42.7 Gy delivered every other day in 2.5 weeks (6.1 Gy per fraction = EQD2Gy: 97.72 Gy_1.5_) is non-inferior to standard conventional fractionation of 78 Gy in 2-Gy fractions over 8 weeks and is specifically relevant for patients with intermediate-risk disease [11]. Many trials [12,13,14,15,16,17,18,19,20,21] including long-term [12] and large cohort [18] outcomes from Western countries confirmed the efficacy of UHF.

The American Society for Radiation Oncology, American Society of Clinical Oncology, and American Urological Association guidelines recommend prescription doses between 35 Gy and 36.25 Gy in five fractions (EQD2 = 85 Gy_1.5_ and 90.6 Gy_1.5_), and doses above 36.25 Gy are not recommended outside the setting of clinical trials [3]. For example, Musunuru et al. reported that 40 Gy/5 fractions elevated toxicity compared with 35 Gy/5 fractions (5% to 24.2% in GU toxicity grade ≥2 and 7.6% to 26.2% in GI toxicity grade ≥2) [19]. Royce et al. reported that at 5 years, patients with low- and intermediate-risk disease, who received an EQD2 of 71 Gy (31.7 Gy in 5 fractions) and an EQD2 of 90 Gy (36.1 Gy in 5 fractions) achieved tumor control probabilities of 90% and 95%, respectively [20]. Our data confirm these findings. The L-UHF arm used EQD2 85–97 Gy_1.5_ and achieved 100% and 97.1% BFFS at 3 years, and the H-UHF group (36 Gy/4 fractions = 108 Gy_1.5_ > 100 Gy_1.5_) showed elevated late toxicity without improved efficacy compared to the DeRT and L-UHF groups. Therefore, at present, according to our findings combined with Western evidence [7,11,12,13,14,15,16,17,18,19,20,21,22,23], we believe that L-UHF (EQD < 100 Gy_1.5_) is a feasible UHF schedule.

We thought that there is room for dose escalation for high-risk groups because of the assumption of a low α/ß ratio of 1.5 Gy for prostate cancer. We hope to improve tumor control while maintaining low levels of late toxicity using a large-fraction dose [16,17,18]. Unfortunately, current UHF clinical trials only support non-inferiority rather than superiority over conventional fractionation [11]. Royce et al. estimated the improvement of tumor control probability up to 95% using high EQD2 of 102 Gy (38.7 Gy in five fractions) compared with 90% when EQD2 of 97 Gy (37.6 Gy in five fractions) was used [20]. Although the biochemical control rate did not improve as per the literature and our findings [2,3,22], Zelefsky et al. reported positive biopsy rates of 47.6%, 19.2%, 16.7%, and 7.7% after 32.5 Gy, 35 Gy, 37.5 Gy, and 40 Gy in five fractions, respectively, which suggested the importance of dose escalation [24]. In addition, although late toxicity was higher than with L-UHF and DeRT, the frequency of toxicity grade ≥3 in the H-UHF group was only 2% in GI and 1% in GU (8% and 10% grade 2), which could be comparable to other studies [12,16,20,21]. Many trials showed similar or higher GI [15,16] and GU toxicity [12,21,25,26]. For instance, Zimmerman et al. reported grade 3–4 toxicity 12.5% in GI and 3.8% in GU (17.5% and 27.5% grade 2) in a phase II trial [26], and Kishan et al. reported 0.4% GI and 1.8% GU (4.5% and 11.2% grade 2) in a large cohort including 2143 patients [18]. Therefore, we believe that there is room to elevate the dose with meticulous caution for toxicity in high-risk prostate cancer.

For the delivery of SBRT, there are several radiation techniques used to deliver a large-fraction dose to the prostate [27,28,29,30]. RT modality, as provided by the robot-assisted technique Cyber Knife^®^ (CK), which can deliver such radiation within a high-fraction dose, is currently witnessing increased usage in the treatment of prostate cancer with low to intermediate risk [27,28,29,30]. IMRT techniques, including rotational approaches as helical tomotherapy and volumetric modulated arc therapy (VMAT), can also deliver a high daily fraction with high conformity and reduce the dose to the surrounding healthy tissue. Chen et al. reported that, based on physical dosimetry and radiobiologic considerations, helical tomotherapy may have advantages over CK, specifically in rectal sparing, which could translate into the clinical benefit of decreased late toxicities [30]. The series of studies showing good dosimetric quality for several SBRT techniques for the treatment of localized prostate cancer was demonstrated [27,28,29,30].

This study has several limitations. First, its retrospective nature, limited follow-up time (especially for the UHF group), and the small sample size may limit its application. We admitted our efficacy data for UHF were not conclusive but suggestive. Second, other predisposing factors should be discussed, including prescribed dose, meticulous dosimetric factors (V46Gy, etc.) [20], and non-dosimetric factors (prostate and irradiated volume, preexisting symptoms or surgery (Transurethral resection of the prostate, etc.), anticoagulant usage, daily versus every other day irradiation, etc.) [31]. Third, although using a free database is beneficial, retrospective databases may not record toxicity and tumor control outcomes and may rather be ambiguous according to the schedule of heterogeneous follow-up periods.

## 5. Conclusions

UHF showed equivalent outcomes to DeRT, although not conclusive but suggestive due to the short follow-up period of the UHF arm. As L-UHF showed lower toxicity than H-UHF, L-UHF using EQD2 ≤ 100 Gy_1.5_ is a feasible UHF schedule with a good balance between toxicity and efficacy.

## Figures and Tables

**Table 1:** Patients’ characteristics between ultrahypofrationated and conventional to medium hypofractionated radiotherapy.

Variables	Group	DeRT	UHF	*p*-Value
		(*n* = 499)	(*n* = 253)	
Age		71.0 [51.0, 86.0]	72.00 [54.0, 86.0]	0.069
iPSA (mg/mL)		10.21 [3.00, 1454.0]	8.12 [1.70, 188.0]	**<0.001**
T (%)	≤T2a	250 (50.2)	178 (70.4)	**<0.001**
	T2b or c	121 (24.3)	50 (19.8)	
	T3≤	127 (25.5)	25 (9.9)	
GS (%)	≤6	118 (23.6)	56 (22.1)	**<0.001**
	7	202 (40.5)	140 (55.3)	
	8≤	178 (35.7)	57 (22.5)	
	NA	1 (0.2)	0 (0.0)	
NCCN (%)	High	271 (54.3)	67 (26.5)	**<0.001**
	Intermediate	172 (34.5)	153 (60.5)	
	Low	56 (11.2)	33 (13.0)	
EQD2Gy (α/β = 1.5 Gy)	(Gy_1.5_)	78.0 [72.0, 91.5]	90.6 [85.0, 108.0]	**<0.001**
Follow-up periods	(months)	61.7 [9.0, 96.0]	32.0 [22.0, 97.0]	**<0.001**
Hormonal therapy (%)	Yes	343 (68.7)	149 (58.9)	**0.009**
	No	156 (31.3)	104 (41.1)	
Total Hormonal therapy duration	(months)	8.00 [2.0, 96.0]	12.0 [2.0, 51.0]	0.749
Neoadjuvant Hormonal therapy (%)	Yes	332 (83.8)	143 (56.5)	**<0.001**
	No	64 (16.2)	110 (43.5)	
Neoadjuvant duration	(months)	6.00 [1.0, 96.0]	6.00 [1.0, 48.0]	**<0.001**
Adjuvant Hormonal therapy (%)	Yes	108 (21.7)	81 (32.0)	**0.002**
	No	390 (78.3)	172 (68.0)	
Adjuvant duration	(months)	23.50 [3.0, 8000]	23.0 [1.0, 33.0]	0.199

Data are presented as patients’ number (%) or median [range] values; Bold values indicate statistical significance. EQD2Gy = n × d ([α/β] + d)/([α/β] + 2); *n* = Number of treatment fractions; d = Dose per fraction in Gy, α/β = 1.5 Gy. DeRT = dose escalated radiotherapy; UHF = ultrahypofractionated radiotherapy.

**Table 2:** Detailed treatment schedule.

Group	Subgroup	Prescribed Dose/ Fraction No	Treatment	PTNO	EQD2
UHF	L-UHF	35 Gy/5 fr	SBRT	63	85
		32 Gy/4 fr	SBRT	9	87
		36.2 5Gy/5 fr	SBRT	81	91
		34 Gy/4 fr	SBRT	9	97
	H-UHF	36 Gy/4 fr	SBRT	91	108
DeRT		62 Gy/20 fr	IMRT	4	82
		65 Gy/26 fr	IMRT	3	74
		67.5 Gy/27 fr	IMRT	6	77
		70 Gy/28 fr	IMRT	83	80
		72 Gy/36 fr	IMRT	25	72
		72. 6 Gy/33 fr	IMRT	23	77
		74 Gy/37 fr	IMRT	125	74
		74.8 Gy/34 fr	IMRT	103	79
		76 Gy/28 fr	IMRT	1	92
		78 Gy/39 fr	IMRT	81	78
		80 Gy/40 fr	IMRT	45	80

DeRT = dose escalated radiotherapy, UHF = ultrahypofractionated radiotherapy, H-UHF = high-dose UHF (EQD2 > 100 Gy_1.5_), L-UHF = low-dose UHF (EQD2 ≤ 100 Gy_1.5_), fr = fractions, EQD2 = n × d ([α/β] + d)/([α/β] + 2) where *n* = number of treatment fractions, d = dose per fraction in Gy, α/β = 1.5 Gy. Koontz et al. reported that EQD2 > 100 Gy_1.5_ might cause high rates of >Grade 2 toxicities [9] and Ishiyama et al. confirmed those results [4]. Therefore, we examined the impact of this threshold not only on toxicity but also on the efficacy of UHF and DeRT, dividing UHF into two subgroups: H-UHF and L-UHF groups, using a cut-off value of EQD2 = 100 Gy_1.5_.

**Table 3:** Late toxicity.

**Comparison between UHF and DeRT.**						
Toxicities	Grade	UHF		DeRT		*p*-value
		(*n* = 253)		(*n* = 499)		
		No. PT	(%)	No. PT	(%)	
Gastrointestinal	0	206	(81%)	422	(85%)	0.633
	1	36	(14%)	55	(11%)	
	2	9	(4%)	17	(3%)	
	3	2	(1%)	5	(1%)	
Genitourinary	0	170	(67%)	405	(81%)	**0.0001**
	1	69	(27%)	70	(14%)	
	2	13	(5%)	22	(4%)	
	3	1	(0.4%)	2	(0%)	
**Comparison between H-UHF and L-UHF.**						
Toxicities	Grade	L-UHF		H-UHF		*p*-value
		(*n* = 162)		(*n* = 91)		
		No. PT	(%)	No. PT	(%)	
Gastrointestinal	0	151	(93%)	55	(60%)	**<0.0001**
	1	9	(6%)	27	(30%)	
	2	2	(1%)	7	(8%)	
	3			2	(2%)	
Genitourinary	0	131	(81%)	39	(43%)	**<0.0001**
	1	27	(17%)	42	(46%)	
	2	4	(2%)	9	(10%)	
	3			1	(1%)	

UHF = ultrahypofractionated radiotherapy, L-UHF = EQD2 ≤ 100 Gy_1.5_, H-UHF = EQD2 > 100 Gy_1.5_, Bold values indicate statistically significance.

**Table 4:** Uni- and Multi-variate analyses for biochemical control rate using Cox proportional hazards model.

Variable	Strata	Uni-Variate Analysis			Multivariate Analysis		
		HR	95% CI	*p*	HR	95% CI	*p*
Age, years	≤74	1	(referent)	-	1	(referent)	-
	75≤	0.8974	0.5015–1.606	0.7156	0.92	0.51–1.67	0.79
T classification	≤2	1	(referent)	-	1	(referent)	-
	3≤	2.045	1.173–3.566	**0.01164**	1.84	0.94–3.59	0.075
Gleason score	≤7	1	(referent)	-	1	(referent)	-
	8≤	1.682	0.9871–2.867	0.05585	1.6	0.89–2.89	0.12
Pretreatment PSA (ng/mL)	≤10	1	(referent)	-	1	(referent)	-
	10<	2.339	1.342–4.078	**0.002729**	2.3	1.24–4.28	**0.0084**
NCCN risk classification	Low	1	(referent)	-	NA		
	Intermediate	2.03	0.597–6.906	0.257			
	High	3.495	1.073–11.386	**0.0379**			
Hormonal therapy	No	1	(referent)	-	1	(referent)	-
	Yes	0.9285	0.5344–1.613	0.7925	0.45	0.23–0.90	**0.023**
Treatment modalities	DeRT	1	(referent)	-	1	(referent)	-
	UHF	0.6195	0.3079–1.247	0.1797	0.79	0.38–1.63	0.52

Bold values indicate statistically significance.

## Data Availability

The data of UHF and part of EBRT for this manuscript can be obtained from the public database [6] and another part of EBRT can be obtained from the author upon reasonable request.

## Supplementary Materials

The following supporting information can be downloaded at: https://www.mdpi.com/article/10.3390/cancers14010195/s1. Table S1. Patients’ characteristics between ultrahypofrationated and conventional to medium hypofractionated radiotherapy after propensity score matching. Table S2. Patient characteristics between H-UHF and L-UHF groups.
